# Physiological ischemic training improves cardiac function through the attenuation of cardiomyocyte apoptosis and the activation of the vagus nerve in chronic heart failure

**DOI:** 10.3389/fnins.2023.1174455

**Published:** 2023-04-20

**Authors:** Xiuhua Zhu, Shenrui Wang, Yihui Cheng, Hongmei Gu, Xiu Zhang, Meiling Teng, Yingjie Zhang, Jiayue Wang, Wenjie Hua, Xiao Lu

**Affiliations:** ^1^Department of Rehabilitation Medicine, The First Affiliated Hospital of Nanjing Medical University, Nanjing, China; ^2^Department of Cardiology, Nantong Geriatric Rehabilitation Hospital, Branch of Affiliated Hospital of Nantong University, Nantong, China; ^3^Department of Rehabilitation Medicine, West China Hospital, Sichuan University, Chengdu, China; ^4^Children’s Healthcare Department, Nanjing Maternity and Child Health Care Hospital, Nanjing, China

**Keywords:** chronic heart failure, physiological ischemic training, cardiac function, apoptosis, vagus nerve, muscarinic M_2_ receptor

## Abstract

**Purpose:**

This study investigated the functional outcomes of patients with chronic heart failure (CHF) after physiological ischemic training (PIT), identified the optimal PIT protocol, evaluated its cardioprotective effects and explored the underlying neural mechanisms.

**Methods:**

Patients with CHF were randomly divided into experimental group (*n* = 25, PIT intervention + regular treatment) and control group (*n* = 25, regular treatment). The outcomes included the left ventricular ejection fraction (LVEF), brain natriuretic peptide (BNP) and cardiopulmonary parameters. LVEF and cardiac biomarkers in CHF rats after various PIT treatments (different in intensity, frequency, and course of treatment) were measured to identify the optimal PIT protocol. The effect of PIT on cardiomyocyte programmed cell death was investigated by western blot, flow cytometry and fluorescent staining. The neural mechanism involved in PIT-induced cardioprotective effect was assessed by stimulation of the vagus nerve and muscarinic M_2_ receptor in CHF rats.

**Results:**

LVEF and VO_2_max increased while BNP decreased in patients subjected to PIT. The optimal PIT protocol in CHF rats was composed of five cycles of 5 min ischemia followed by 5 min reperfusion on remote limbs for 8 weeks. LVEF and cardiac biomarker levels were significantly improved, and cardiomyocyte apoptosis was inhibited. However, these cardioprotective effects disappeared after subjecting CHF rats to vagotomy or muscarinic M_2_ receptor inhibition.

**Conclusion:**

PIT improved functional outcomes in CHF patients. The optimal PIT protocol required appropriate intensity, reasonable frequency, and adequate treatment course. Under these conditions, improvement of cardiac function in CHF was confirmed through cardiomyocyte apoptosis reduction and vagus nerve activation.

## 1. Introduction

Chronic heart failure (CHF) is a complex clinical syndrome with symptoms (breathlessness, fatigue, and ankle swelling) and signs resulting from a structural or functional impairment of the heart, representing the end stage of various cardiovascular diseases (McDonagh et al., 2021; Writing Committee and Members, 2022). It is one of leading causes of hospitalizations among the elderly, which places a considerable burden on patients, their families and the healthcare system (Van Spall et al., 2019; Agarwal et al., 2021). Therefore, it is of utmost importance to explore efficient and accessible therapies for the growing population with CHF. Despite multiple therapies, such as implantable cardioverter defibrillators, cardiac resynchronization, and orthotopic heart transplantation, the survival after the onset of heart failure is miserably poor, with 5-year mortality rates as high as 46% (Tsao et al., 2022). Moreover, these interventions are expensive or cause other problems, e.g., excessive waiting time, during which some CHF patients may become too weak to undergo transplantation or even die while on the waiting list (Rana et al., 2015). Therefore, the investigation for new interventions to improve the functional recovery of patients is needed.

Physiological ischemic training (PIT) has recently emerged as a novel therapeutic method to achieve protection in the target organ (e.g., heart) by brief repetitive cycles of ischemic training on remote limbs (Ni et al., 2015). Previous studies reported that PIT improves the endothelial function and coronary microcirculation (Kimura et al., 2007; Kono et al., 2014), increases the expression of peripheral blood vascular endothelial growth factor (Gao et al., 2018), and modifies the inflammatory response (Shimizu et al., 2010) in patients with heart failure. However, the effects of PIT on the functional outcomes in CHF patients remain uncertain. Recently, two clinical trials in patients with CHF suggested that PIT does not improve left ventricular performance (Pryds et al., 2017), nor does it have any beneficial effect on the exercise capacity (Groennebaek et al., 2019). This is in somewhat on contradiction with the findings of another previous study, where an improvement in cardiac function was observed in patients with CHF after PIT (Chen et al., 2018). The discrepancy between these studies may be the result of different frequency or course of PIT. Hence, the optimal PIT protocol and its real cardioprotective effects should be investigated.

Apart from that, the understanding of the cardioprotective mechanisms regulating PIT is also important for a further customization of the training protocol. According to a literature review, programmed cell death (PCD) has been reported in the context of PIT. One recent study showed that PIT attenuates acute myocardial injury through the inhibition of apoptosis and the promotion of autophagy (Xu et al., 2022). The cardioprotective effects of PIT in CHF may also be due to modifications of PCD. However, it is not clear how the protective messages from the ischemic limb reached the cardiomyocytes, consequently regulating PCD. Autonomic nervous system imbalance, simplified as increase in sympathetic activity and withdrawal of parasympathetic activity, is involved in the progression of CHF (Florea and Cohn, 2014; van Bilsen et al., 2017). A previous study revealed an impaired vagus nerve activity in the early stage of heart failure, and this decrease in vagal function is associated with poor outcomes in patients with CHF (Bibevski and Dunlap, 2011). Recently, one study demonstrated that PIT attenuates the inhibitory inputs of the dorsal motor nucleus of the vagus, thus modulating cardioprotection (Gorky et al., 2021). These findings suggest that the activation of the vagus nerve may be involved in the cardioprotective effects of PIT against CHF.

Therefore, in this work PIT was performed in patients with CHF, and the changes in functional outcomes after PIT were evaluated. Various PIT cycles at different intensity, frequency, and course were performed in CHF rats to discover the optimal PIT protocol. Once the optimal PIT protocol was determined, the effects on various types of PCD were investigated. Last, we constructed several experiments to explore underlying neural mechanisms of PIT-induced cardioprotective effects in CHF.

## 2. Materials and methods

### 2.1. Human study

#### 2.1.1. Patients

A total of 167 CHF patients were recruited from 2020 to 2022 at the First Affiliated Hospital of Nanjing Medical University to investigate the clinical effects exerted by PIT in patients with CHF. The inclusion criteria were the following: (1) patients aged 18–80 years; (2) patients diagnosed with CHF resulting from coronary artery disease (CAD), confirmed by imaging examination; (3) patients belonging to the functional class II-III according to the New York Heart Association (NYHA); (4) patients eligible for cardiopulmonary exercise test (CPET). The exclusion criteria were the following: (1) acute coronary syndrome; (2) hemodynamically significant valve disease; (3) second- or third-degree atrioventricular block; (4) atrial fibrillation or flutter in the previous 3 months; (5) severe renal or hepatic failure; (6) uncontrolled diabetes and hypertension; (7) acute stroke; (8) peripheral artery disease or deep vein thrombosis; (9) other diseases impeding the participation of patients. Fifty eligible CHF patients were enrolled and provided the written informed consent. All the enrolled patients received a standard pharmaceutical treatment (including antiplatelet drugs, statin, and agents for controlling comorbidities), as well as adequate health education (covering basic knowledge of CHF, symptoms monitoring, medication adherence, diet guidance and lifestyle intervention). One self-checklist was developed to ensure all patients follow the standard pharmaceutical treatment, routine physical activity and dietary habits, without great modification during the study. The general conditions of the patients are listed in Supplementary Table S1. There was no significant difference between the two groups at baseline. Computer-generated random number table was used according to simple randomization. The fifty eligible CHF patients were randomly divided into experimental group (*n* = 25) who underwent supplementary PIT and control group (*n* = 25) without supplementary PIT.

#### 2.1.2. PIT in CHF patients

Standard instructions regarding PIT were provided to patients in the experimental group. Three cycles of 5 min of ischemia were applied to the upper left arm, which was achieved by the inflation of the blood-pressure cuff to 200 mmHg, followed by 5 min of reperfusion when the cuff was deflated, according to a previous study (Cunniffe et al., 2017). During the study, patients were instructed to perform PIT once daily at home over a duration of 12 weeks. To ensure consistency of PIT, all patients were given the same instruction of PIT and one research assistant conducted teleconsultations with patients once per week to confirm all patients performed PIT correctly. The patients in the control group were not subjected to PIT.

#### 2.1.3. Outcomes in CHF patients

Primary outcomes included left ventricular ejection fraction (LVEF), left ventricular end-systolic diameter (LVESD), and left ventricular end-diastole diameter (LVEDD), using echocardiography, measured as indicators of cardiac function. The secondary outcomes were the following: (1) BNP level in peripheral blood; (2) maximal oxygen uptake (VO_2_max) and anaerobic threshold (AT) measured using CPET; (3) 6-min walk test (6MWT), (4) quality of life assessed by the Minnesota Living with Heart Failure Questionnaire (MLHFQ) (Morcillo et al., 2007).

### 2.2. Animal study

#### 2.2.1. Rat model and PIT cycles

A total of 90 male Wistar rats 6–8 weeks old and weighing 200 ± 20 g were randomly divided into sham operation (SO), or CHF group. According to previous studies (Samsamshariat et al., 2005; Sehirli et al., 2013), CHF was induced by the ligation of the left anterior descending (LAD) coronary artery. Briefly, thoracotomy was performed in the rats, then LAD was permanently ligated at 1–2 mm below the junction of the pulmonary artery conus and left atrial appendage. SO rats were subjected to the same procedure without LAD ligation. After 4 weeks of LAD ligation, LVEF lower than 50% by echocardiography was the successful sign of CHF model. Additionally, to ensure the establishment of CHF model, we also identified hypertrophy and fibrotic response in heart tissue *via* Wheat germ agglutinin (WGA) staining and Masson trichrome staining in the exploratory preliminary experiment (results can be seen in Supplementary Figure S1). PIT was performed with a tourniquet to induce physiological ischemia and reperfusion on remote limbs in CHF rats, as described in previous studies (Zheng et al., 2014; Ni et al., 2015; Zheng et al., 2017).

Table 1 describes the investigation to find the optimal PIT protocol and the underlying neural mechanisms in CHF rats.

**Table 1:** Assignment of CHF rats.

Group	Description
**Investigation to find the optimal PIT protocol**	
Intensity	
1 min-PIT group	1 min ischemia followed by 1 min reperfusion, five cycles per day, 8 weeks
5 min-PIT group	5 min ischemia followed by 5 min reperfusion, five cycles per day, 8 weeks
10 min-PIT group	10 min ischemia followed by 10 min reperfusion, five cycles per day, 8 weeks
Frequency	
3 cycles-PIT group	5 min ischemia followed by 5 min reperfusion, three cycles per day, 8 weeks
5 cycles-PIT group	5 min ischemia followed by 5 min reperfusion, five cycles per day, 8 weeks
7 cycles-PIT group	5 min ischemia followed by 5 min reperfusion, seven cycles per day, 8 weeks
Course	
4 weeks-PIT group	5 min ischemia followed by 5 min reperfusion, five cycles per day, 4 weeks
8 weeks-PIT group	5 min ischemia followed by 5 min reperfusion, five cycles per day, 8 weeks
12 weeks-PIT group	5 min ischemia followed by 5 min reperfusion, five cycles per day, 12 weeks
**Explorations of underlying neural mechanisms**	
Role of vagus nerve	
VNS group	invasive vagus nerve stimulation (more details in the Supplementary material) performed before the daily PIT
VNC group	Vagus nerve cut at cervical segment (more details in the Supplementary material) performed before PIT treatment
Role of muscarinic M_2_ receptor	
M_2_R+ group	nonselective muscarinic acetylcholine receptor agonist (oxotremorine, 0.2 mg/kg, MCE, Shanghai China) intravenously injected *via* the tail vein before the daily PIT
M_2_R− group	selective muscarinic M_2_ receptor antagonist (methoctramine, 0.5 mg/kg, MCE, Shanghai China) intravenously injected *via* the tail vein before the daily PIT

#### 2.2.2. Cell culture and treatment

H9c2 myoblasts, a cell model used as an alternative for cardiomyocytes, were purchased from Shanghai FuHeng Cell Center (Shanghai, China). They were cultured in Dulbecco’s modified eagle medium (DMEM, Gibico, Waltham, CA, United States) supplemented with 10% fetal bovine serum, 100 U/mL penicillin and 100 U/mL streptomycin and incubated at 37°C under a humidified 5% CO_2_ environment. H9c2 cells were cultured in serum-free DMEM for 24 h and treated with angiotensin II (Ang II, 1 μmol/L, Sigma) for 24 h to induce cell hypertrophy and fibrotic response. Phosphate buffer saline was used as a negative control. After 24 h, the control cells were treated with phosphate buffer saline.

#### 2.2.3. Echocardiography

Transthoracic echocardiography was performed on all rats using Vevo3100 with a MX250S linear array transducer (VisualSonics, Toronto, ON, Canada). Rats were anesthetized using 2–3% isoflurane and they were kept warm on a heated platform (37°C). The chest fur was removed, and a layer of acoustic coupling gel was applied to the thorax. The cardiac function was measured on M-mode images after the alignment in transverse B-mode with papillary muscles. Echocardiography data were collected using VisualSonics Vevo 3,100 and analyzed using Vevo LAB 3.1.0.

#### 2.2.4. Plasma biomarker detection

Three biomarkers were selected, such as NT-proBNP, tumor necrosis factor-α (TNF-α) and interleukin 6 (IL-6), which were measured by enzyme-linked immunosorbent assay (ELISA), as previously described (Steinhorn et al., 2018). ELISA kits were purchased from SBJbio life sciences (Nanjing, China), and were performed according to the manufacturer’s instructions. Absorbance was measured at 450 nm using a microplate reader (Labsystems Multiskan MS, Helsinki, Finland).

#### 2.2.5. Norepinephrine concentration assessment

Plasma NE concentration was determined as an indirect index of sympathetic activity (Kandlikar and Fink, 2011) using a commercial ELISA kit (CSB-E07022r, CUSABIO, Wuhan, China) following the manufacturer’s instructions. The reaction was read at 450 nm using a microplate reader (Labsystems Multiskan MS, Helsinki, Finland).

#### 2.2.6. Acetylcholinesterase activity assay

The activity of AChE, an enzyme involved in parasympathetic neurotransmission, is often used as a potential marker of the parasympathetic activity (Fedorova et al., 2015; Cavalcante et al., 2020). AChE activity in cardiac tissue was measured using the Ellman method (Ellman et al., 1961). Acetylthiocholine iodide was used as substrate and dithionitrobenzoic acid used as color indicator. Finally, AChE activity was monitored at 412 nm.

#### 2.2.7. Western blot

Western blot was performed according to a standard protocol (Li et al., 2018). The primary antibodies used were the following: Cleaved-caspase3 (Abcam, Cambridge, MA, United States), Bcl-2 (Abcam, Cambridge, MA, United States), Bax (Abcam, Cambridge, MA, United States), RIPK1 (Proteintech, Wuhan, China), MLKL (Proteintech, Wuhan, China), SLC7A11 (Proteintech, Wuhan, China), GPX4 (Proteintech, Wuhan, China), LC3 (Proteintech, Wuhan, China), p62 (Proteintech, Wuhan, China), tubulin (Proteintech, Wuhan, China) and GAPDH (Proteintech, Wuhan, China) used a s the loading control.

#### 2.2.8. Measurement of cholinergic neuron density

The histochemical staining of cholinergic neurons was performed using the Karnovsky–Roots method (Karnovsky and Roots, 1964), as previously described. All reagents were purchased from Sigma-Aldrich (St. Louis, MO, United States). Cholinergic neurons were visualized using a light microscope (Leica DM500, Wetzlar, Germany). The density of cholinergic neurons in cardiac tissues was measured using the image-analyzing system Image-pro plus 6.0 (Media Cybernetics, Inc., Rockville, MD, United States).

#### 2.2.9. Flow cytometry

Annexin V-Fluorescein isothiocyanate (FITC) and propidium iodide (PI) staining kit (Vazyme, Nanjing, China) were used to identify apoptotic H9c2 cells. Flow cytometry analysis was performed using Cytoflex (Beckman Coulter, Brea, CA, United States) and data were analyzed using the FlowJo software (TreeStar, SanCarlos, CA, United States). The second quadrant (FITC+/PI+) showed the late apoptotic cells and the fourth quadrant (FITC+/PI−) showed the early apoptotic cells.

#### 2.2.10. YO-PRO-1/PI staining

Double staining using YO-PRO-1/PI kit (Beyotime, Shanghai, China) was used to detect necrotic H9c2 cells and distinguish apoptosis from necrosis. In brief, the dye mix solution (YO-PRO-1 dye and PI) was prepared following the manufacturer’s instructions. Cells were fixed with the above mixed dye solution, wrapped into a tin foil to avoid light and incubated at 37°C for 30 min. A fluorescence inverted microscope was used to visualize apoptotic cells by a green fluorescence, whereas necrotic cells exhibited both red fluorescence and green fluorescence. The fluorescence images were analyzed by a THUNDER Imaging System (Leica Microsystems, Wetzlar, Germany).

#### 2.2.11. Lipid reactive oxygen species assay

Lipid ROS assay was performed using C11-BODIPY581/591-Lipid Peroxidation Sensor (Thermo Fisher Scientific, Waltham, CA, United States) to quantify ferroptosis in H9c2 cells. Briefly, cells were treated with 1 ml C11-BODIPY581/591 dye 10 μM for 30 min at 37°C. Oxidation of C11-BODIPY581/591 was revealed by the change in BODIPY fluorescence from red to green, and fluorescence imaging were acquired using the THUNDER Imaging System (Leica Microsystems, Wetzlar, Germany).

#### 2.2.12. Monodansylcadaverine assay

MDC autophagy staining assay kit (Beyotime, Shanghai, China) was used to measure autophagy in H9c2 cells. Briefly, cells were treated with 50 μM MDC and incubated at 37°C for 15 min in the dark. Cells were washed with phosphate buffer saline and visualized using the THUNDER Imaging System (Leica Microsystems, Wetzlar, Germany), and MDC activity was evaluated by the quantification of green fluorescence.

### 2.3. Statistical analysis

Statistical analysis was performed using GraphPad Prism 9 (GraphPad, San Diego, CA, United States), and SPSS 21.0 (IBM Corp., Armonk, NY, United States). The comparisons between two groups were performed by Student’s *t*-test or Mann–Whitney *U*-test. The comparisons among multiple groups (≥3) were performed by one-way analysis of variance (ANOVA) with Tukey’s *post hoc* test. Continuous variables were presented as mean ± standard deviation or median (25th–75th percentile). Categorical variables were presented as proportions (percentages) and analyzed by chi-square test or Fisher exact test. Spearman’s correlation test was used for correlation analysis. A *p*-value < 0.05 was considered statistically significant.

## 3. Results

### 3.1. Improved functional outcomes after PIT in CHF patients

**Figure 1:**
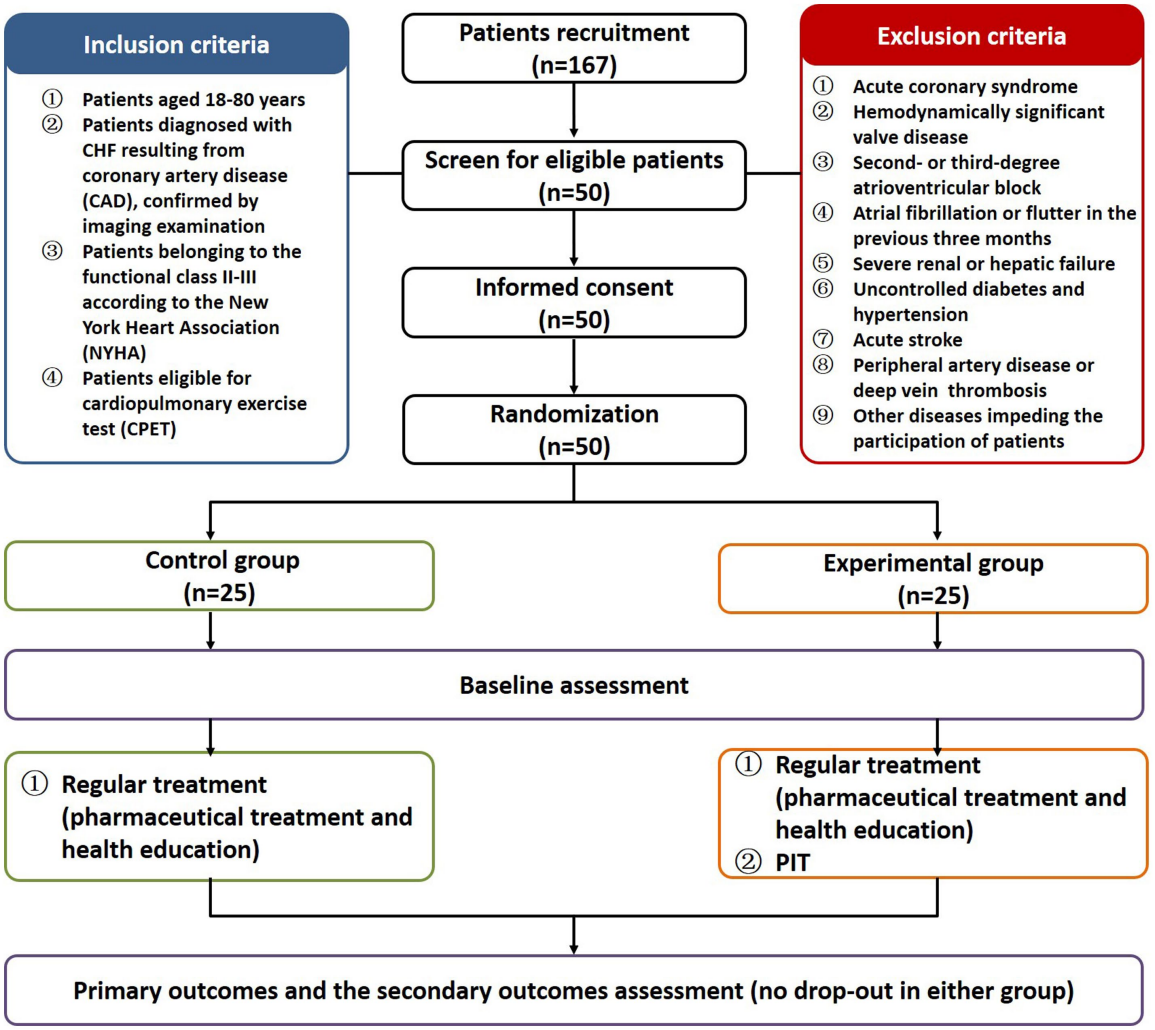
Experimental flow chart.

**Figure 2:**
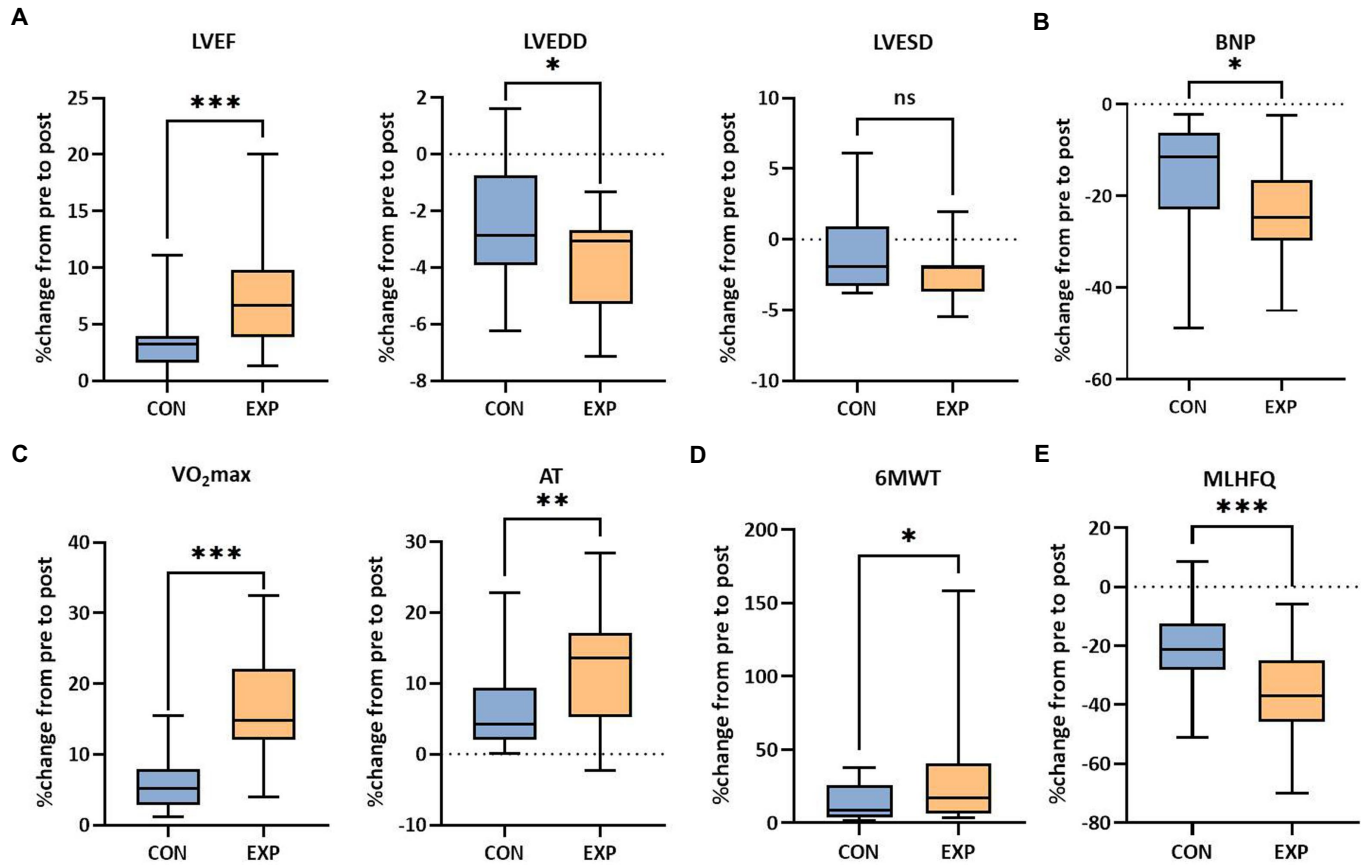
Functional outcomes of the control group and experimental group. **(A)** Cardiac function (LVEF, LVEDD and LVESD). **(B)** Plasma BNP level in peripheral blood. **(C)** Cardiopulmonary fitness (VO_2_max and AT). **(D)** Exercise capacity (distance under the 6MWT). **(E)** Quality of life (MLHFQ scores, lower score indicating higher quality of life). ns, not significant; *p < 0.05, **p < 0.01, and ***p < 0.001. CON, control group (regular treatments, including pharmaceutical treatment and health education); EXP, experimental group (additional PIT intervention after regular treatments).

All patients completed the 12-week treatment and the outcomes were measured before and after the intervention. In total, 50 patients were included in the final analysis with 25 patients in the experimental group and 25 patients in the control group (Figure 1). The raw data of the pre- and post-intervention are listed in Supplementary Table S2, and changes from pre- to post-intervention between groups were analyzed using the non-parametric test. Cardiac function (including LVEF and LVEDD, except LVESD) was significantly improved in the experimental group compared to the control group (6.67 *vs* 3.28% for LVEF, *p* < 0.001; −3.08 *vs* −2.86% for LVEDD, *p* < 0.05; −2.00 *vs* −1.89% for LVESD, Figure 2A). Plasma BNP level was also significantly decreased in the experimental group than in controls (−24.68 *vs* −11.46% for BNP, *p* < 0.05, Figure 2B). Furthermore, the results of CPET and 6MWT indicated that the cardiopulmonary exercise capacity was significantly improved in the experimental group (11.85 *vs* 5.24% for VO_2_max, *p* < 0.001; 13.60 *vs* 6.20% for AT, *p* < 0.01; 17.22 *vs* 8.99% regarding the distance under the 6MWT, *p* < 0.05, Figures 2C,D). The MLHFQ score showed that patients in the experimental group had a higher quality of life than those in the control group (−36.90 *vs* −21.21% for the MLHFQ score, *p* < 0.001, Figure 2E).

### 3.2. Optimal PIT protocol in CHF rats

**Figure 3:**
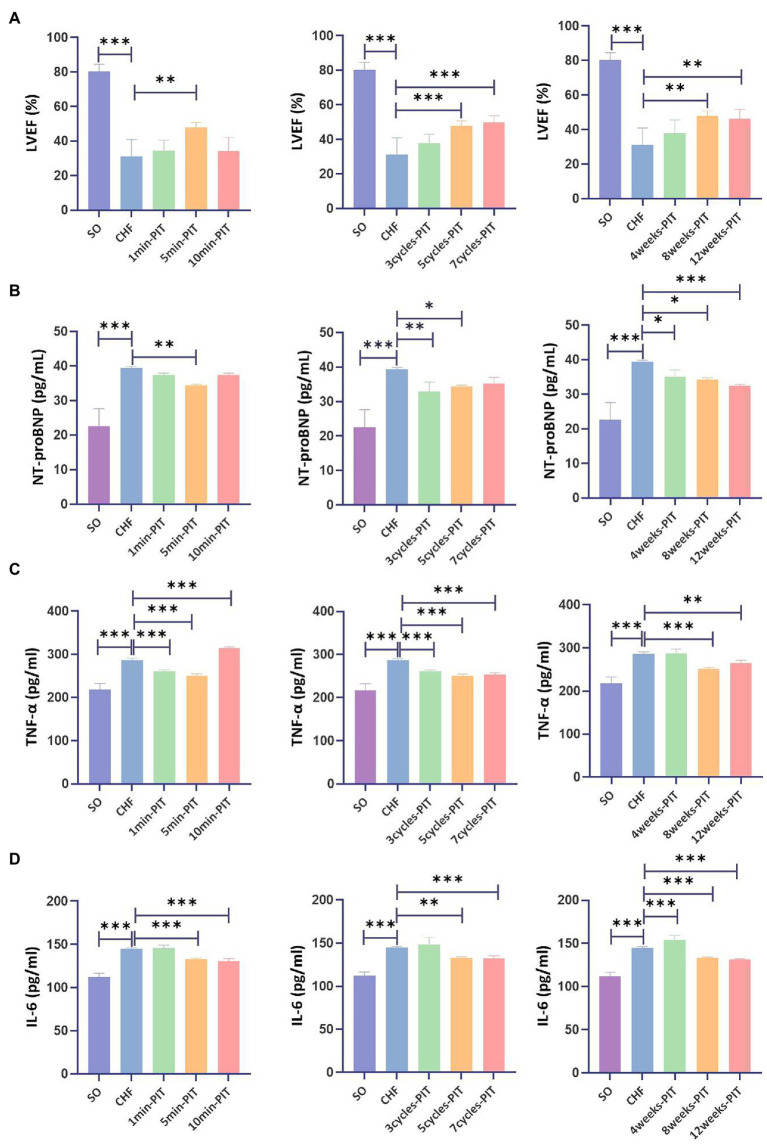
Effects on LVEF and cardiac biomarkers after various PIT protocols in CHF rats. **(A)** Left ventricular ejection fraction (LVEF). **(B)** Plasma NT-proBNP level. **(C)** Plasma tumor necrosis factor-α (TNF-α) level. **(D)** Plasma interleukin 6 (IL-6) level. *p < 0.05, **p < 0.01, and ***p < 0.001. SO, sham operation; CHF, chronic heart failure; PIT, physiological ischemic training.

LVEF in CHF rats treated with 5 min-, 5 cycles-, 7 cycles-, 8 weeks-, and 12 weeks-PIT was significantly improved compared to no-treatment group (Figure 3A). NT-proBNP in CHF rats treated with 5 min-, 3 cycles-, 5 cycles-, 4 weeks-, 8 weeks-, and 12 weeks-PIT was significantly decreased compared to no-treatment group (Figure 3B). TNF-α in CHF rats treated with 1 min-, 5 min-, 3 cycles-, 5 cycles-, 7 cycles-, 8 weeks-, and 12 weeks-PIT was significantly decreased compared to no-treatment group (Figure 3C). IL-6 in CHF rats treated with 5 min-, 10 min-, 5 cycles-, 7 cycles-, 8 weeks-, and 12 weeks-PIT was significantly downregulated compared to no-treatment group (Figure 3D). The combined results of LVEF and cardiac biomarkers (NT-proBNP, TNF-α and IL-6) indicated that CHF rats treated with 5 min-, 5 cycles-, 8 weeks-, and 12 weeks-PIT had a significant improved cardioprotection compared to no-treatment group, while no significant results on LVEF between 8 weeks- and 12 weeks-PIT was observed. Collectively, 5 min ischemia followed by 5 min reperfusion on remote limbs, five cycles per day for 8 weeks, represented the optimal PIT protocol in CHF rats.

### 3.3. PIT-induced reduction of cardiomyocyte apoptosis

**Figure 4:**
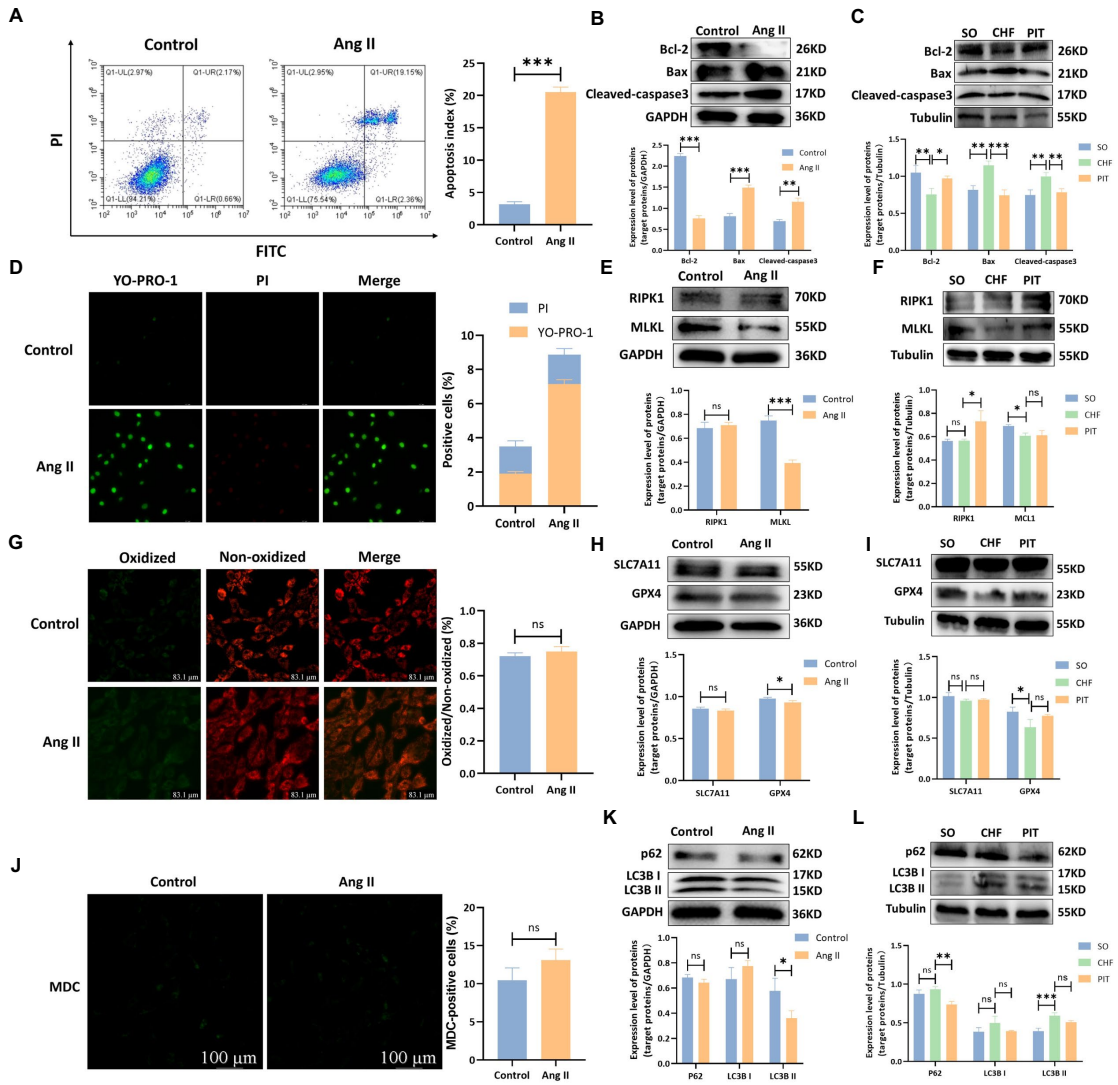
Effects of Ang II and PIT on programmed cell death of H9c2 cells and cardiomyocytes of CHF rats, respectively. **(A)** Apoptosis detected by flow cytometry in control and Ang II treated H9c2 cells. **(B)** Protein expression of Bcl-2, Bax, and Cleaved-caspase3 in control and Ang II treated H9c2 cells. **(C)** Protein expression of Bcl-2, Bax, and Cleaved-caspase3 in cardiomyocytes of SO, CHF and PIT rats. **(D)** Necrosis detected by YO-PRO-1/PI staining in control and Ang II treated H9c2 cells. **(E)** Protein expression of RIPK1 and MLKL in control and Ang II treated H9c2 cells. **(F)** Protein expression of RIPK1 and MLKL in cardiomyocytes of SO, CHF and PIT rats. **(G)** Ferroptosis detected by ROS assay in control and Ang II treated H9c2 cells. **(H)** Protein expression of SLC7A11 and GPX4 in control and Ang II treated H9c2 cells. **(I)** Protein expression of SLC7A11 and GPX4 in cardiomyocytes of SO, CHF and PIT rats. **(J)** Autophagy detected by MDC assay in control and Ang II treated H9c2 cells. **(K)** Protein expression of p62 and LC3B in control and Ang II treated H9c2 cells. **(L)** Protein expression of p62 and LC3B in cardiomyocytes of SO, CHF and PIT rats.

Various types of PCD, including apoptosis, necrosis, ferroptosis and autophagy, were detected *in vitro* and *in vivo*. Apoptotic index of H9c2 cells were significantly increased after the treatment with Ang II compared with the index in the control group (Figure 4A). Bcl-2 expression was decreased, while Bax and Cleaved-caspase3 increased in H9c2 cells treated with Ang II compared with the expression in the control group (Figure 4B). *In vivo*, Bcl-2 expression was decreased, Bax and Cleaved-caspase3 were increased in cardiomyocytes of CHF group compared to that in the SO group. However, Bcl-2 expression was increased, Bax and Cleaved-caspase3 expression were decreased in cardiomyocytes of PIT group compared to that in the CHF group (Figure 4C). Additionally, necrosis, ferroptosis and autophagy were measured (Figures 4D,G,J) as well as RIPK1 and MLKL expression (Figures 4E,F), SLC7A11 and GPX4 expression (Figures 4H,I), and p62 and LC3B expression (Figures 4K,L). However, the levels of necrosis, ferroptosis and autophagy were slightly changed in Ang II-treated H9c2 cells compared to the control group, although without being significant, and the expression level of proteins was lack of consistent changes in H9c2 cells treated with Ang II and cardiomyocytes of SO, CHF, and PIT rats.

### 3.4. Neural mechanisms regulating the cardioprotective effects of PIT in CHF rats

#### 3.4.1. PIT-induced upregulation of the vagal activity in CHF rats.

**Figure 5:**
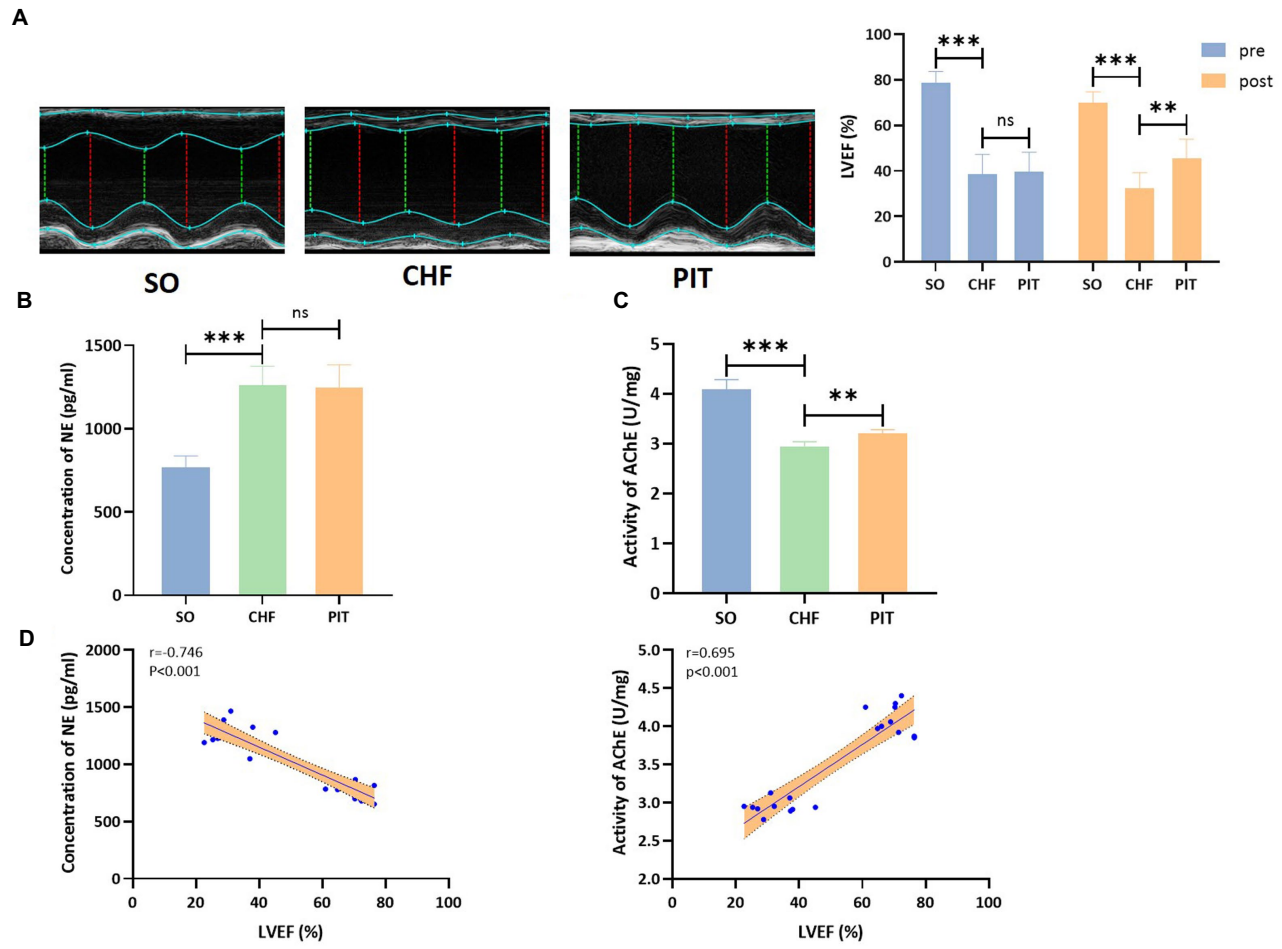
Effects of PIT on autonomic nervous activity in CHF rats. **(A)** Representative echocardiography images and quantitative results of LVEF. **(B)** Concentration of NE in circulation. **(C)** Activity of AChE in cardiac tissues. **(D)** Correlation between LVEF and NE concentration as wells AChE activity. Correlation coefficients and *p* values were obtained from Spearman’s correlation analysis. ns, not significant; **p < 0.01 and ***p < 0.001. SO, sham operation; CHF, chronic heart failure; PIT, physiological ischemic training.

LVEF was significantly increased in the PIT group than in the CHF group (Figure 5A). Then, the evaluation of the sympathetic and parasympathetic activity revealed that the concentration of NE in circulation was significantly increased in the CHF group compared to the SO group, while no significant effect was observed between the CHF and PIT group (Figure 5B). However, AChE activity in cardiac tissues was decreased in the CHF group compared to the SO group, and increased in the PIT group compared to the CHF group (Figure 5C). Furthermore, LVEF was significantly negatively correlated with NE concentration, while positively correlated with AChE activity (Figure 5D). Thus, our speculation was that the vagal activity upregulated by PIT in CHF rats was associated with improved cardiac function.

#### 3.4.2. PIT induced cardioprotective effect through the activation of the vagus nerve in CHF rats

**Figure 6:**
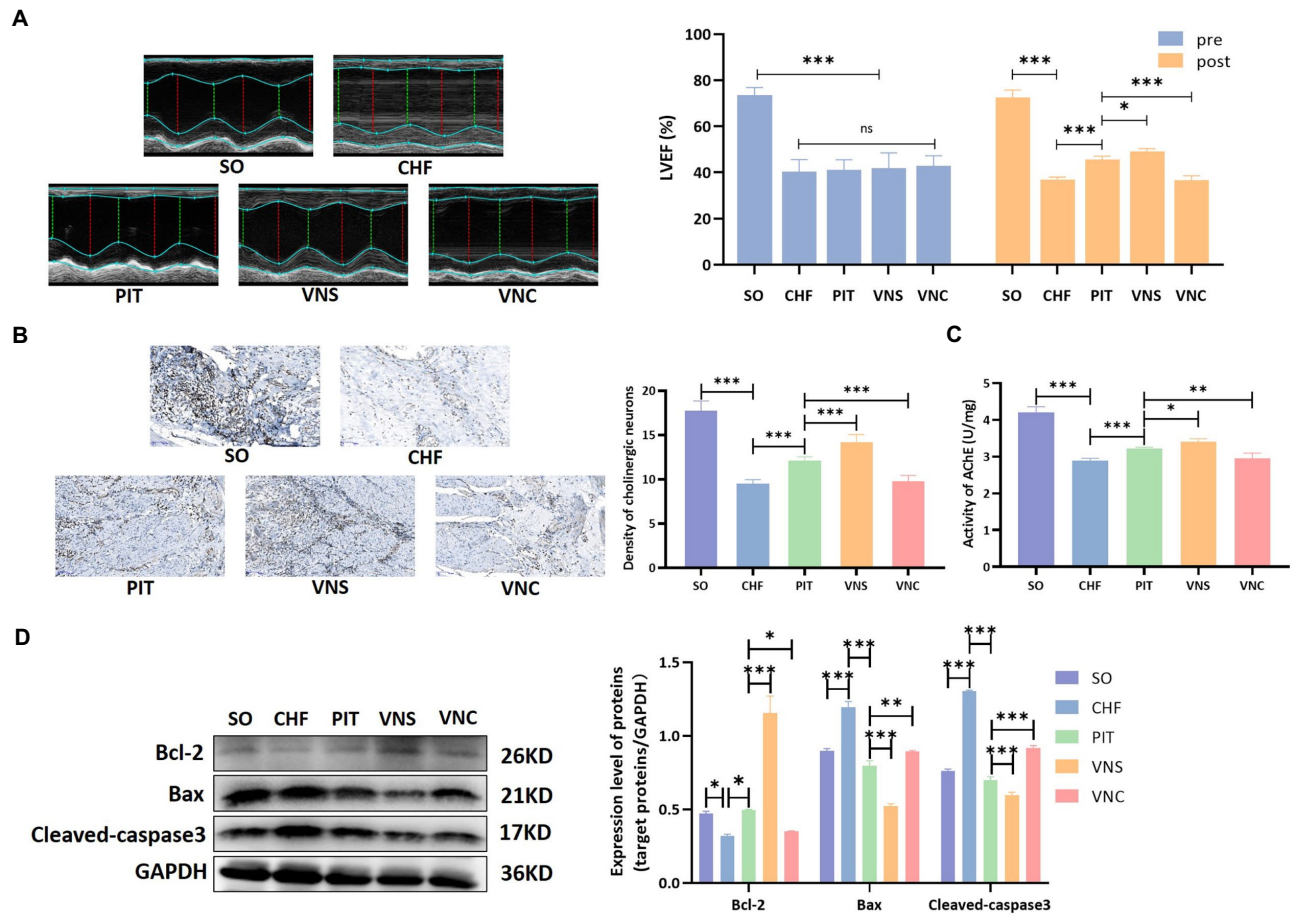
Cardioprotective effect of PIT under vagus nerve stimulation and cut. **(A)** Representative echocardiography images and quantitative results of LVEF. **(B)** Distribution of cholinergic neurons in cardiac tissues by Karnovsky–Roots staining, and density of cholinergic neurons. **(C)** AChE activity in cardiac tissues. **(D)** Bcl-2, Bax, and Cleaved-caspase3 expression by western blot. *p < 0.05, **p < 0.01, and ***p < 0.001. SO, sham operation; CHF, chronic heart failure; PIT, physiological ischemic training; VNS, vagus nerve stimulation; VNC, vagus nerve cut.

The improvement of LVEF induced by PIT increased in the vagus nerve stimulation (VNS) group, while decreased in the vagus nerve cut (VNC) group compared to the PIT group (Figure 6A). The density of cholinergic neurons in cardiac tissues was decreased in the CHF group compared to that in the SO group, increased in the PIT group compared to that in the CHF group, increased in the VNS group compared to that in the PIT group, while decreased in the VNC group compared to that in the PIT group (Figure 6B). Additionally, similar trends were observed for AChE activity in cardiac tissues (Figure 6C). Bcl-2 expression was decreased in the CHF group compared to that in the SO group, increased in the PIT group compared to that in the CHF group, while increased in the VNS group and decreased in the VNC group compared to that in the PIT group. However, Cleaved-caspase3 and Bax expression showed an opposite change trend (Figure 6D). Collectively, our results suggested that the improvement of LVEF and the inhibition of cardiomyocyte apoptosis induced by PIT in CHF rats were mediated by the activation of the vagus nerve.

#### 3.4.3. PIT induced cardioprotective effect depending on the recruitment of muscarinic M_2_ receptor in CHF rats

**Figure 7:**
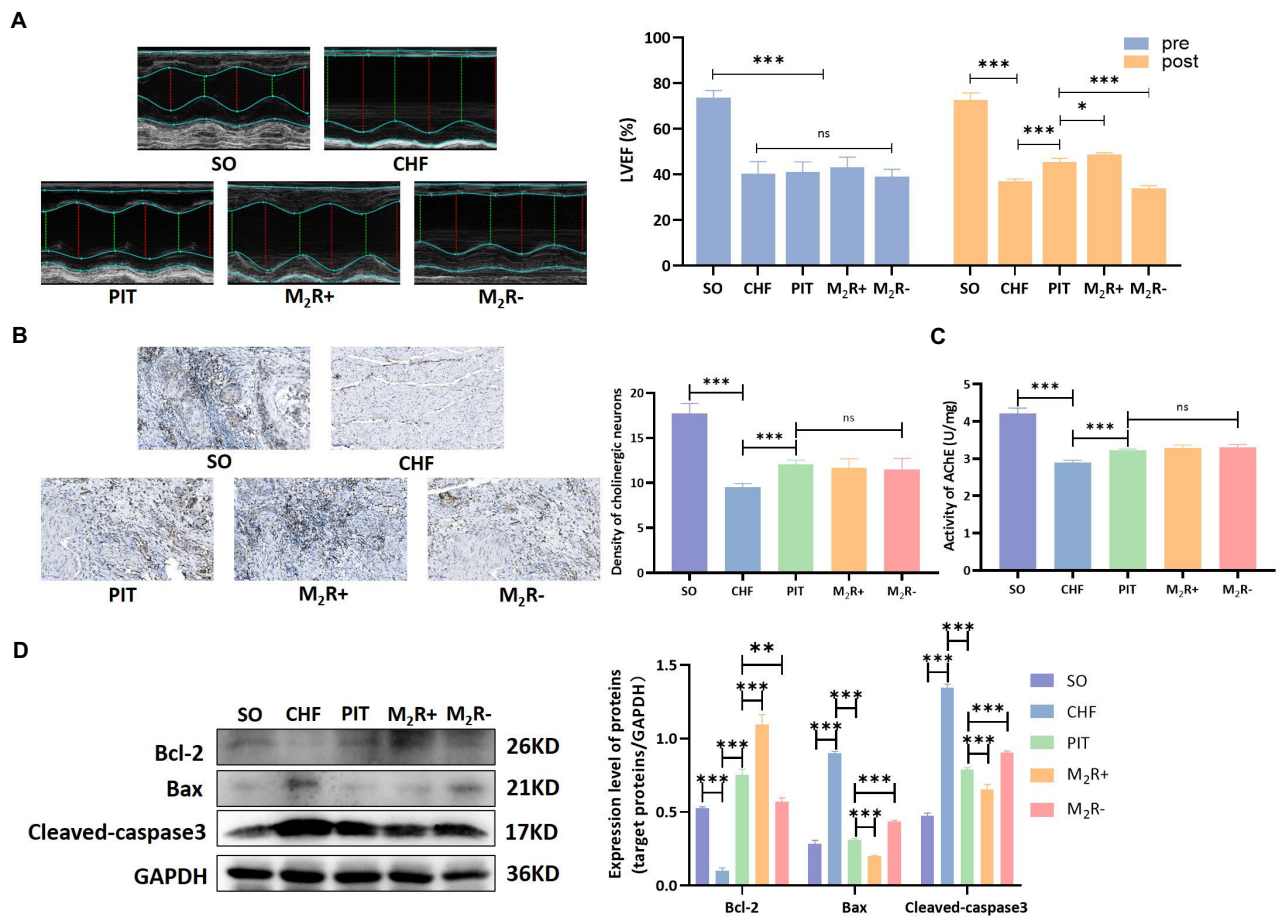
Cardioprotective effect of PIT under muscarinic M_2_ receptor activation and inhibition. **(A)** Representative echocardiography images and quantitative results of LVEF. **(B)** Distribution of cholinergic neurons in cardiac tissues by Karnovsky and Roots staining, and density of cholinergic neurons. **(C)** AChE activity in cardiac tissues. **(D)** Proteins related to apoptosis by western blot. *p < 0.05, **p < 0.01, and ***p < 0.001. SO, sham operation; CHF, chronic heart failure; PIT, physiological ischemic training; M_2_R+, M_2_ receptor activation; M_2_R−, M_2_ receptor inhibition.

Acetylcholine, the classical neurotransmitter released from the vagus nerve, regulates cardiac functions mainly depending on the activation of muscarinic receptors (Brodde et al., 2001). In the heart, muscarinic M_2_ receptor is the typical functional subtype, we observed that intravenous injection of nonselective muscarinic acetylcholine receptor agonist (oxotremorine, 0.2 mg/kg/day for 4 weeks) *via* the tail vein in CHF rats exerted protective effects in the heart, however, application of selective muscarinic M_2_ receptor antagonist (methoctramine, 0.5 mg/kg/day for 4 weeks) *via* tail intravenous injection in CHF rats had no significant effects on cardiac performance (Supplementary Figure S2). Further, the role of muscarinic M_2_ receptor recruitment in PIT induced cardioprotection was evaluated in the condition of muscarinic M_2_ receptor agonist (M_2_R+) and antagonist (M_2_R−). LVEF improvement was observed in the PIT group, while the improvement was increased in the M_2_R+ group and decreased in the M_2_R− group compared to that in the PIT group (Figure 7A). The density of cholinergic neurons and AChE activity were not statistically different among PIT, M_2_R+ and M_2_R− group (Figures 7B,C). Bcl-2 expression was decreased in the CHF group compared to that in the SO group, increased in the PIT group compared to that in the CHF group, was increased in the M_2_R+ group and decreased in the M_2_R− group compared to that in the PIT group (Figure 7D). However, the expression of Cleaved-caspase 3 and Bax, which was increased in the CHF group compared to that in the SO group, and decreased in the PIT group compared to that in the CHF group, was decreased in the M_2_R+ group and increased in the M_2_R− group compared to that in the PIT group (Figure 7D). Hence, our hypothesis was that the recruitment of muscarinic M_2_ receptor was necessary for PIT-induced cardioprotective effects in CHF rats, while the inhibition of the M_2_ receptor diminished these protective effects.

## 4. Discussion

The current study demonstrated that PIT produced beneficial effects on the functional outcomes of patients with CHF, including the improvement of cardiac function, cardiopulmonary fitness, exercise capacity, and quality of life. Then the optimal PIT protocol was identified; it was composed of five cycles of 5 min ischemia with 5 min reperfusion on remote limbs for 8 weeks. The use of the optimal PIT protocol resulted in the reduction of cardiomyocyte apoptosis in CHF rats after PIT. Finally, PIT induction of cardioprotective effects in CHF was confirmed through the activation of the vagus nerve.

Our human study revealed that PIT produced a significant increase of LVEF and LVEDD in CHF. The decrease of LVESD was not significant, which could be explained by changes of LVEDD occurred at an early stage, while LVESD at a later stage (Abdel-Bary et al., 2019). Furthermore, the reduction of the left ventricular dysfunction following PIT might contribute to a great cardiopulmonary capacity and quality of life. Decreased plasma BNP after PIT also demonstrated cardioprotection in CHF. All these findings suggested that PIT improved the functional outcomes of CHF patients. Some contradictory statements in previous studies might be due to the short course of the treatment, e.g., PIT was performed for only 28 ± 4 days in a previous study (Pryds et al., 2017). Another study pointed out that the intensity and frequency of PIT are also correlated with the degree of cardioprotective effects (Barbosa et al., 1996). This might be attributed to the discrepancy in PIT training protocol adopted in different studies. Thus, the exploration of the optimal PIT training protocol is necessary for a consensus on the cardioprotective effects of PIT.

Based on the above findings, various PIT regimes were applied in CHF model rats. These PIT training protocols varied in intensity, frequency, and course. LVEF and cardiac biomarkers (NT-proBNP, TNF-α and IL-6) levels were evaluated. The combined results showed that the PIT protocol with five cycles of 5 min ischemia with 5 min reperfusion on remote limbs for 8 weeks was the optimal protocol, since it resulted in a significant improvement of LVEF, and reduction of adverse cardiac biomarkers in CHF rats. Thus, the improvement of cardiac function in CHF was established based on this optimal protocol. However, the changes at a cellular level responsible for these functional improvements were unclear.

PCD is an important adaptive mechanism in response to various physiological or pathological conditions. The aberrant activation of PCD is associated with the progressive loss of cardiomyocytes during the progression of heart failure, leading to a deterioration of the cardiac function and adverse cardiac remodeling (Zhou et al., 2021). PCD includes several subtypes, including apoptosis, necrosis, ferroptosis and autophagy. The results in our study indicated that apoptosis was the main form of cardiomyocyte death in the pathophysiology of CHF. Apoptosis, a highly regulated biological process, is a common contributor for the decrease of cardiac function in heart failure (van Empel et al., 2005; Zhang et al., 2019). The expression of proteins (Cleaved-caspase3, Bcl-2, Bax) in the current study revealed that PIT inhibited cardiomyocyte apoptosis, which was responsible for the increased cardiac ejection fraction in CHF rats. Nonetheless, the specific mechanisms regulating this apoptosis were not well understood yet.

The imbalance of the autonomic nervous system is involved in the pathophysiology of heart failure (Florea and Cohn, 2014; Zannad, 2019). Our study found that PIT did not affect the sympathetic activity but upregulated the parasympathetic activity in CHF. Thus, a CHF model with vagus nerve stimulation or vagotomy was constructed in rats to further investigate whether the increase in vagal activity was indispensable for PIT-induced cardioprotective effects in CHF. Our observations revealed that the upregulation of the vagal activity accompanied with the improvement of LVEF and the inhibition of cardiomyocyte apoptosis were enhanced under vagus nerve stimulation, while these changes were inhibited under vagotomy. These results confirmed that PIT induced cardioprotective effects in CHF depending on the activation of the vagus nerve. Acetylcholine, which is released from postganglionic sites following vagal stimulation, functions by activating the muscarinic acetylcholine receptor (mAChR) in the cardiomyocyte membrane (Coote, 2013). The mAChR family consists of five distinct subtypes, named M_1_ to M_5_ (Kruse et al., 2014). Three of these receptor subtypes (M_1_, M_2_, and M_3_) are the main functional subtypes distributed within the heart (Saternos et al., 2018). The current study demonstrated that the intravenous administration of oxotremorine (a nonselective mAChR agonist) in CHF rats induced an improvement of the cardioprotective effects of PIT, while the administration of methoctramine (the selective M_2_ receptor antagonist) abrogated the protective effects. However, a previous study suggested that the activation of the muscarinic M_3_ receptor was crucially important for PIT-induced cardioprotection, based on the abolishment of the cardioprotection after M_3_ receptor inhibition (Basalay et al., 2016). Both the current and previous studies used only one selective muscarinic receptor blocker targeting M_2_ or M_3_ subtype; therefore, a role of other mAChR subtypes could not be excluded. The involvement of other signaling pathways might also contribute to the final benefits of PIT on functional recovery although needs to be further explored with advanced techniques and well-designed studies.

## 5. Conclusion

In conclusion, an improvement of functional outcomes after PIT treatment was observed in patients with CHF. The optimal protocol was composed of five cycles of 5 min ischemia with 5 min reperfusion on remote limbs for 8 weeks, which significantly improved cardiac ejection fraction and inhibited cardiomyocyte apoptosis. All these cardioprotective effects were generated through the activation of the vagus nerve.

## Data availability statement

The original contributions presented in the study are included in the article/Supplementary material, further inquiries can be directed to the corresponding author.

## Ethics statement

The studies involving human participants were reviewed and approved by the Research Ethics Committee at Jiangsu Province Hospital, the First Affiliated Hospital of Nanjing Medical University. The patients/participants provided their written informed consent to participate in this study. The animal study was reviewed and approved by Institutional Animal Care and Use Committee of Nanjing Medical University.

## Author contributions

XHZ and XL designed the study. SW and HG recruited CHF patients and performed CPET and 6MWT tests. YC, XiZ, MT, and JW built rat CHF models and performed PIT treatments. XHZ, YZ, and SW conducted various measurements *in vivo* and vitro. XHZ, WH, and YC performed statistical analyses. XHZ drafted the manuscript and all authors participated in the modification of the manuscript. All authors contributed to the article and approved the submitted version.

## Funding

This work was supported by the National Natural Science Foundation of China (grant numbers: 81772441 and 82072546).

## Conflict of interest

The authors declare that the research was conducted in the absence of any commercial or financial relationships that could be construed as a potential conflict of interest.

## Publisher’s note

All claims expressed in this article are solely those of the authors and do not necessarily represent those of their affiliated organizations, or those of the publisher, the editors and the reviewers. Any product that may be evaluated in this article, or claim that may be made by its manufacturer, is not guaranteed or endorsed by the publisher.
