# Supplementary material for: Physiological ischemic training improves cardiac function through the attenuation of cardiomyocyte apoptosis and the activation of the vagus nerve in chronic heart failure
Source: Front Neurosci. 2023 Apr 20;17:1174455. doi: 10.3389/fnins.2023.1174455 (PMC10157045; doi:10.3389/fnins.2023.1174455)
Supplement: Supplementary file 1 [file Data_Sheet_1.docx]

**Supplementary Materials**

**Methods**

**Models of Vagus Nerve Stimulation (VNS):** Vagus nerve stimulating electrodes were prepared according to the following method. Electrode tubes were constructed from two 1.5 cm sections of pure silver wire (0.25 mm diameter) covered with polyethylene tubing (PE-10). Each section was soldered to a 30-gallon. Then two 10 cm silver-plated stranded wires were inserted into a 3.5 mm section of polyethylene tubing (PE-160) to keep the electrodes stable and separated (1.5 mm). All seams and open ends were sealed with Epoxy. Prior to surgery, a 3 mm long strip of tubing was stripped from the inner side of each PE-10 tube to expose a segment of the silver wire for contact with the vagus nerve. Each vagus nerve stimulating electrode was sterilized prior to being implanted in the rat. To implant vagus nerve stimulating electrodes, rats were anesthetized, shaved in head and neck, and fixed onto the operating table in supine position. Then a 1 cm incision was made on the right ventral side of the neck just lateral to the midline, the layer of subcutaneous fat and underlying muscles were bluntly dissected, and the sternohyoid and sternomastoid muscles were separated longitudinally to reveal the right vagus nerve which lies lateral to the carotid artery within the carotid sheath. A 5 mm length of the vagus nerve was exposed, and the prepared vagus nerve stimulating electrode was tightly coiled around the nerve in a helical fashion. Effective contact with the exposed vagus nerve and electrodes was verified with a continuity test using an ohmmeter. In order to prevent displacement of the nerve or electrode, the electrode was loosely sutured to the mediodorsal surface of the sternomastoid muscle. The electrode leads were threaded through the fascia to the dorsal aspect of the neck and out of the incision. The incision was sutured and the leaving 3cm length of leads folded and placed on the dorsal side of the head and neck. One week following the surgery, rats with CHF received VNS (frequency 20 Hz, pulse width 0.5 ms, duty cycle 10 s on to 50 s off) on the right vagus nerve for continuous 20 min before the daily PIT. The effectiveness of VNS was checked by hemodynamic responses in each rat, that heart rate decreased by 20–30 bpm without great changes in the arterial pressure in rats with CHF during VNS.

**Models of Vagus Nerve Cut (VNC):** Rats with CHF received vagus nerve cut at cervical segment before PIT. The cervical vagotomy was performed according to the following method. Briefly, after anesthesia, rats were fixed onto the operating table in supine position, and a 1 cm incision was made in the middle of neck. Then the tissue was separated layer by layer, the left and right cervical vagal nerves were isolated from carotid sheath and cut off, and the incision was sutured.


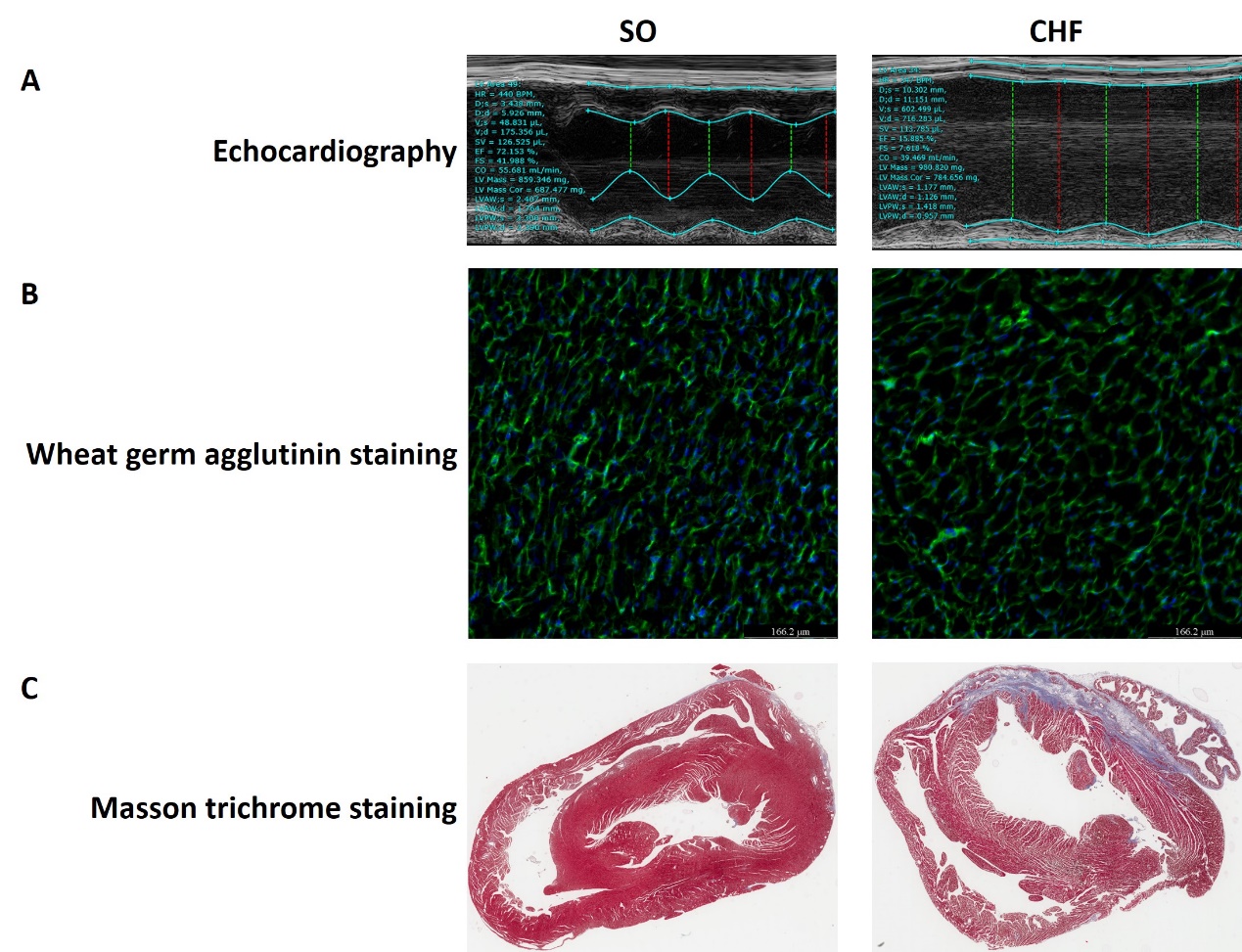


**Figure S1 Establishment of CHF model** (A)Transthoracic echocardiography demonstrates decreased cardiac function in CHF as compared to SO. (B) Wheat germ agglutinin staining shows cardiomyocyte hypertrophy in CHF as compared to SO. (C) Masson trichrome staining shows myocardial fibrosis in CHF as compared to SO.


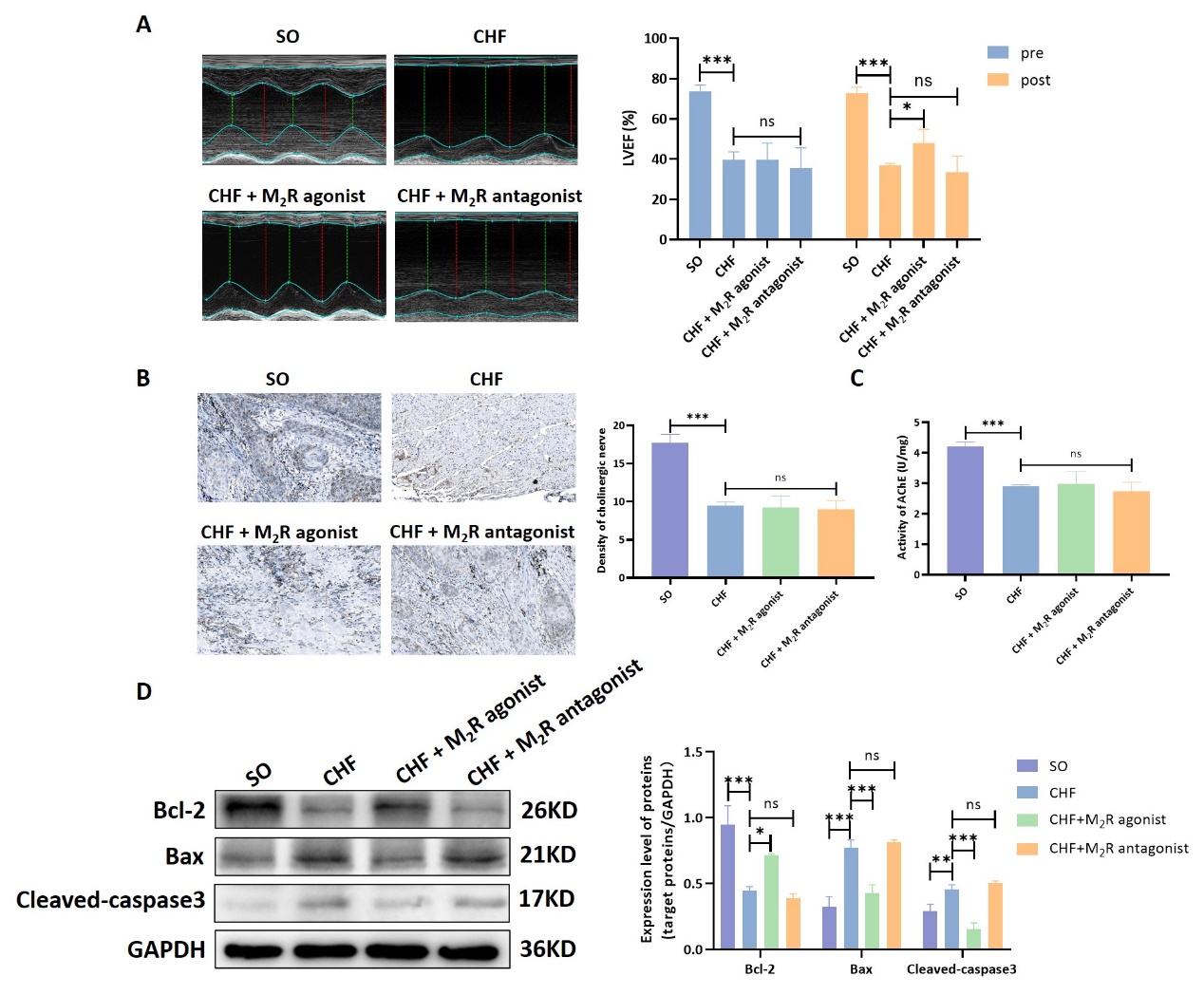


**Figure S2 Effects of M_2_R agonist and M_2_R antagonist in CHF group** (A) Representative echocardiography images and quantitative results of LVEF. (B) Distribution of cholinergic neurons in cardiac tissues by Karnovsky and Roots staining, and density of cholinergic neurons. (C) AChE activity in cardiac tissues. (D) Proteins related to apoptosis by western blot. ns, not significant, **P* < 0.05, ***P* < 0.01 and ****P* < 0.001. SO, sham operation; CHF, chronic heart failure; PIT, physiological ischemic training.

**Table S1 Baseline of the demographic and clinical characteristics of the enrolled patients.**

|  | **CON group (n = 25)** | **EXP group (n = 25)** | **P value** |
| --- | --- | --- | --- |
| **Age (year)** | 67.7±6.0 | 68.5±6.1 | 0.64 |
| **Male** | 11/25 (44%) | 12/25 (48%) | 0.78 |
| **Height (cm)** | 165.3±4.6 | 166.2±5.3 | 0.52 |
| **Weight (kg)** | 74.2± 7.5 | 73.3±6.5 | 0.65 |
| **Mean heart rate (beats/min)** | 76±5 | 78±6 | 0.21 |
| **Blood pressure at randomization** |  |  |  |
| **SBP (mmHg)** | 126±7.3 | 127±8.2 | 0.65 |
| **DBP (mmHg)** | 77.6± 5.2 | 78.2± 6.5 | 0.72 |
| **LVEF (%)** | 58.44±6.92 | 56.64±8.40 | 0.41 |
| **NYHA classification** |  |  |  |
| **II** | 21/25 (84%) | 21/25 (84%) | 1 |
| **III** | 4/25 (16%) | 4/25 (16%) | 1 |
| **Medical history** |  |  |  |
| **Coronary artery disease** | 25/25 (100%) | 25/25 (100%) | 1 |
| **Hypertension** | 18/25 (72%) | 18/25 (72%) | 1 |
| **Diabetes** | 8/25 (32%) | 7/25 (28%) | 0.76 |
| **PCI history** | 2/25 (8%) | 1/25 (4%) | 0.55 |
| **Medication** |  |  |  |
| **Antiplatelet agents** | 25/25 (100%) | 25/25 (100%) | 1 |
| **Statins** | 25/25 (100%) | 25/25 (100%) | 1 |
| **Diuretics** | 15/25 (60%) | 16/25 (64%) | 0.77 |
| **ACEI/ARB/** **ARNI** | 12/25 (48%) | 13/25 (52%) | 0.78 |
| **Beta blockers** | 16/25 (64%) | 18/25 (72%) | 0.54 |
| **Calcium antagonists** | 18/25 (72%) | 17 /25 (68%) | 0.76 |
| **Oral antidiabetic agents** | 5/25 (20%) | 4/25 (16%) | 0.71 |
| **GLP-1 receptor agonists** | 1/25 (4%) | 1/25 (4%) | 1 |
| **Insulin** | 5/25 (20%) | 5/25 (20%) | 1 |

Data are presented as mean ± standard deviation or proportion (%).

Abbreviations: CON, control group; EXP, experimental group; SBP, systolic blood pressure; DBP, diastolic blood pressure; LVEF, left ventricular ejection fraction; NYHA, New York heart association; PCI, percutaneous transluminal coronary intervention; ACEI/ARB/ARNI, angiotensin-converting enzyme inhibitor/angiotensin receptor blocker/angiotensin receptor neprilysin inhibitor; GLP-1, glucagon-like peptide-1.

**Table S2 Pre-intervention and post-intervention data.**

|  | **Pre-intervention** | | **Post-intervention** | |
| --- | --- | --- | --- | --- |
|  | **Con** | **Exp** | **Con** | **Exp** |
| **LVEF (%)** | 58.44±6.92 | 56.64±8.40 | 60..28±6.84 | 60.52±7,40 |
| **LVEDD (mm)** | 66.64±4.99 | 67.32±4.47 | 65.08±4.23 | 64.75±4.60 |
| **LVESD (mm)** | 55.76±4.93 | 56.44±4.42 | 55.12±4.29 | 54.76±4.32 |
| **BNP (pg/ml)** | 188 (142.5,277) | 230 (132.3,315) | 165 (121,236) | 180 (110,210.5) |
| **VO_2_max (mL/kg/min)** | 13.56 (12.56,17.15) | 13.23 (11.57,16.46) | 14.86 (13.20,17.66) | 15.62 (13.96,19.01) |
| **AT (mL/kg/min)** | 10.45 (9.08,13.02) | 9.99 (8.40,13.96) | 10.48 (9.47,13.47) | 11.21 (10.06,14.67) |
| **6MWT (m)** | 372.0±128.35 | 344.48±134.10 | 408.76±115.08 | 413.8±100.61 |
| **MLHFQ (score)** | 58 (50,67.5) | 64(53.5,73.5) | 48 (39,54) | 41(28.3,53.5) |

Data were presented as mean ± standard deviation or median (25th–75th percentile).

Abbreviations: LVEF, left ventricular ejection fraction; LVEDD, left ventricular end-diastole diameter; LVESD, left ventricular end-systolic diameter; BNP, brain natriuretic peptide; VO2max, maximal oxygen uptake; AT, anaerobic threshold; 6MWT, six-minute walk test; MLHFQ, Minnesota Living with Heart Failure Questionnaire.
